# Vernal Keratoconjunctivitis: an update focused on clinical grading system

**DOI:** 10.1186/s13052-019-0656-4

**Published:** 2019-05-21

**Authors:** A. M. Zicari, G. Capata, M. Nebbioso, G. De Castro, F. Midulla, L. Leonardi, L. Loffredo, A. Spalice, L. Perri, M. Duse

**Affiliations:** 1 Department of Pediatrics, “Sapienza” University of Rome, Viale Regina Elena 324, Rome, Italy; 2 Department of Internal Medicine and Medical Specialities, “Sapienza” University of Rome, Rome, Italy; 3 Department of Sense Organs, “Sapienza” University of Rome, Rome, Italy

**Keywords:** Vernal keratoconjunctivitis, Grading, Diagnosis, Clinical markers

## Abstract

**Introduction:**

Vernal keratoconjunctivitis (VKC) is a severe disease with a prevalence of < 1 case out of 10,000 in Europe, which occurs mainly in pediatric age and is characterized by a severe and often bilateral chronic inflammation of the ocular surface. The diagnosis is generally confirmed by the finding at the ocular examination of conjunctival hyperemia, papillary hypertrophy in the tarsal conjunctiva, giant papillae, papillae in the limbus region.

**Objective:**

Aim of this review is to provide an updated overview on the disease focused on clinical grading system, searching papers published in the last decade on VKC in scientific databases.

**Results:**

Currently there are no standardized criteria for diagnosis of VKC and there is no uniformity to define disease severity, which makes difficult to diagnose and treat the disease.

**Conclusions:**

Given the wide overlap of the symptoms of VKC with the allergic conjunctivitis, criteria of probable, possible or improbable diagnosis are needed, providing pediatricians with parameters useful for deciding whether to drive the patient to the ophthalmologist for diagnostic confirmation.

## Background

Vernal keratoconjunctivitis (VKC) is characterized by a severe and often bilateral chronic inflammation of the ocular surface, which can result in permanent injury if not adequately recognized and treated [1]. It is a rare disease with a prevalence of < 1 case out of 10,000 in Europe, which occurs mainly in pediatric age and resolves spontaneously after puberty [2, 3]. VKC follows a typical seasonal pattern with onset in spring, exacerbation in summer and a tendency to remission in the autumn-winter period [3]. Criteria of diagnosis are still not well defined and specific criteria of suspicion of the disease are lacking too. Therefore, VKC remains underestimated and can lead to complications with irreversible damages. A unique and widely shared grading of its severity is lacking too, the choice of the better therapy remains still arbitrary [4]: a worldwide consensus is awaited.

We looked for articles investigating clinical and diagnostic tools that could be potentially useful to manage Vernal Keratoconjunctivitis in everyday clinical practice. The objective of this review is to provide an updated overview on the disease with a focus on clinical grading systems implemented until now.

## Methods of research

We searched Pubmed database and Cochrane library with the term “vernal keratoconjunctivitis” AND “clinical score”, “vernal keratoconjunctivitis” AND “scoring”, “vernal keratoconjunctivitis” AND “grading”. All studies published from January 1, 2007 through January 22, 2019 were included for a total of 200 citations. We excluded articles in languages other than English, Italian and French. The reference list of the selected paper was manually screened to identify any additional references not found directly on the electronic databases. The search provided 17 relevant papers.

### Overview on VKC

VKC begins typically in the first decade of life and resolves spontaneously after puberty even though in the most severe cases it can leave permanent lesions. The disease affects males more frequently than females, with a ratio of 2–3: 1 and it is prevalent in dry hot climates, specifically at the Mediterranean basin, the Middle East, Central and West Africa, India, and South America [5–9]. Despite the typically seasonal trend, perennial forms are reported with exacerbations in the spring-summer period [3].

The clinical picture of VKC is characterized by ocular symptoms such as ocular pruritus, tearing, burning, foreign body sensation and photophobia [3]. Photophobia is a troublesome symptom that worsens the quality of life, limits daily activities and is exacerbated by exposure to light sources or screens, making the use of sunglasses indispensable. At the eyelid eversion, hyperemia of the bulbar and tarsal conjunctiva may be observed with the presence of papillae of variable size and occasionally of gelatinous infiltrates in the limbus region (Trantas Horner nodules). Just the finding of papillae at the limbus and/or tarsal level, strongly suggestive of VKC, allows differentiating it from seasonal or perennial allergic conjunctivitis [3, 10]. Occasionally, in particular during the exacerbations, corneal involvement may occur with punctate keratitis, macro-erosions and shield ulcers that must be promptly treated to prevent permanent outcomes [11].

The complications occur mainly on the tarsal conjunctiva and the limbus area; rarely the cornea may be affected too [3, 12]. The cornea damages are due to the combination of the mechanical injury caused by the friction of the giant papillae of the conjunctiva on the corneal epithelium with the releasing of inflammatory mediators from activated eosinophils and mast cells infiltrated into the conjunctiva [13, 14]. As a result, shield ulcers and plaques may develop and cause a permanent reduction in visual acuity up to 6% of all patients [15, 16]. These chronic lesions can lead to further complications such as microbial keratitis, amblyopia, and rarely to corneal perforation [17]. Microbial keratitis is one of the most severe complications and it is due to the greater susceptibility to infection of the VKC eye.

The rate of infections of eyes with shield ulcers ranges about 9–10%; *Staphylococcus epidermidis* and Streptococcus pneumonia are the most frequent bacteria isolated, followed by Corynebacterium species, Neisseria meningitides, Klebsiella pneumonia, Brevibacterium species; occasionally fungal infections from Aspergillus were reported in patients with VKC [18].

Keratoconus is another severe complication of VKC and affect about 15% of patients; in a large population from Italy, a frequency of around 2% has been observed [19, 20].

Pathogenesis: The development of VKC is the result of complex interactions between genetic, environmental and immunologic factors. The association of VKC with specific HLA haplotype has been few investigated with inconsistent results [21–23]. We observed in a limited number of VKC children the high expression of some haplotypes, such as DQB1*05, but the case study was too small to give meaningful indications and merely suggested that VKC may be a syndrome with a probable genetic predisposition not yet well defined [24].

Many signs, symptoms and histological studies suggest that an IgE-mediated inflammation plays the major role in the pathogenesis of VKC. About half of VKC patients are also allergic, their ocular symptoms worse at the allergen exposure and cytological pattern in tears and tissues support the role of specific IgE–mast cell activation in the development of the disease [25–28]. Moreover, specific IgE are detectable in serum and in tears, at least in the active phase of the disease. Leonardi et al. detected IgE using ImmunoCAP ISAC microarray in lacrimal samples of 10 VKC patients: six resulted to have specific IgE in tears but only three of them had specific IgE detectable in the serum [29]. Notably, the severity score of the disease was not correlated to the presence of secretory or serum IgE. However, also non-IgE mediated mechanism could be involved.

Increased numbers of CD4+ Th2 lymphocytes in the conjunctiva and overexpression of cytokines and costimulatory molecules are well documented [30–34]. VKC patients overexpress both Th2 and Th1-derived cytokines, pro-inflammatory molecules, chemokines and growth factors as expression of a real inflammatory storm [35, 36].

Also IL-17 contributes to inflammation and it is implicated in many autoimmune and allergic diseases. As for the eye, its implication has been demonstrated in scleritis, uveitis, dry eye disease, and inflammation of the cornea due to Herpes virus. We studied the production of IL-17 in a limited number of VKC children and found that serum level of IL-17 was significantly higher respect to the healthy controls although it did not correlate with clinical grading of severity [37]. A subsequent study confirmed our results in larger VKC population and showed, unlike our results, that the levels of IL-17 correlated with the severity, in particular with the presence and size of Trantas dots [38].

A high percentage (30.8%) of VKC children present antinuclear antibodies (ANA positivity) and an even greater percentage (about 50%) have a familiar history of autoimmune disorders, suggesting that VKC could be a bridge disease between systemic autoimmune disorders and atopic condition [39, 40].

Moreover, VKC seems to be not an isolated disease of the eye but rather a systemic inflammatory response related to traffic of inflammatory cells and mediators from outside and inside the eye as suggested by recent observations. As we recently reported, inflammatory proteins such as high-mobility group box-1 (HMGB1) and soluble receptor for advanced glycation end products (sRAGE) are both increased in the serum of children with VKC [41]. Their concentration correlate with severity of disease, being higher during the acute phase of the disease and decreasing in remission or during local therapy. Notably, these inflammatory molecules do not differ between VKC children atopic and non-atopic as well as between ANA-positive and ANA-negative children [41]. Caputo et al. dosed HMGB-1 in lacrimal fluid samples obtained from VKC children and from healthy subjects [42]. They confirmed our results, demonstrating significantly higher levels of HMGB-1 in tears collected from VKC children. Interestingly, lacrimal amount of HMGB-1 correlated with clinical score by Bonini.

These data as whole suggest that VKC is in any case a systemic inflammatory disease, regardless of whether it is driven by a shifted Th1 or Th2 immune response, but no one of these markers of inflammation are available for routine clinical practice.

### Grading of VKC

Although the literature shows that the clinical picture of VKC and its complications are well defined, there is not currently a unique criterion of suspicion. In view of the wide overlap of the symptoms of VKC with the allergic conjunctivitis, the criteria of probable, possible or improbable diagnosis - as for example the presence of major symptoms (and how many) and minor symptoms - are needed, providing pediatricians with parameters useful for deciding whether to drive the patient to the ophthalmologist for diagnostic confirmation.

The finding of papillary hyperplasia is mandatory for the diagnosis of VKC and there is agreement on the classification of the disease based on the part of conjunctiva involved (Bulbar/Limbal, Palpebral and Mixed form) [43]. Papillae are variable in size ranging from 0.1 to 5 mm in diameter but there is no unanimous threshold measure that distinguishes giants from small ones, since cut off proposed range from 3 mm to 1 mm in diameter, as recently suggested [3].

As regards the severity of the disease, instead, some models of combined evaluation of the single symptoms have been proposed.

Pucci et al. [27] developed a grading score of subjective ocular symptoms (itching, photophobia, tearing, foreign body and burning sensation) ranging from zero (no symptoms) to 15 (severe clinical picture). For each symptom, the score assigned was 1: mild symptoms of discomfort just noticeable, 2: moderate discomfort for most of the day without interfering with daily routine activities, 3: severe symptoms disrupting daily routine activities and forcing the patient to stay at home most of the time.

Spadavecchia et al. proposed to evaluate patients according to a combination of two disease severity scales, one based on classical ocular signs such as conjunctival hyperemia, small or giant papillae, Trantas, and the other one based on subjective ocular symptoms such as itching, photophobia tearing, burning and foreign body sensation. These scales were graded from zero (absent) to two (severe) and children were classified as having severe VKC if the score was ≥3 points for each scale [28].

More recently, Bonini et al. [44] suggest a new grading system based on the clinical characteristics of VKC. Patients were defined as Grade 0 (quiescent) when they are free of symptoms. Papillae may be present without local signs of disease activity (no conjunctival hyperemia). As Grade 1 (mild intermittent), when the patients refer onset of symptom during spring season and present a slight ocular inflammation (mild hyperemia) without corneal involvement, giant papillae may be present. As Grade 2 (moderate intermittent/persistent), when patients are presenting the same symptoms as in grade 1 but more frequent and disturbing during the day, with mild to severe papillary reaction and conjunctival hyperemia. The intermittent form defines patients with occasional symptoms without corneal involvement; the persistent variant includes patients symptomatic every day during season with occasional involvement of the cornea (superficial punctate keratitis). As Grade 3 (severe), if symptoms are present every day and hamper daily activities, severe conjunctival hyperemia and secretion may be associated to the presence of Horner-Trantas dots and the cornea may present superficial punctate keratitis, papillary reaction is moderate to severe. As Grade 4 (very severe) if severe itching and photophobia are present everyday with mucus discharge on the ocular surface and between papillae, Horner-Trantas dots are present and corneal complications are common. As Grade 5 (evolution) when the patients present occasional symptoms during seasonal periods, conjunctival papillary reaction may be present, but the cornea is spared and conjunctival fibrosis may be seen on the upper tarsal conjunctiva or at the fornix.

In 2009 Shoji et al. proposed another clinical grading score for ocular allergic disease named 5–5-5 exacerbation grading scale, consisting of the following 3 graded groups of clinical observations: 1- the 100-point-grade group (100 points for each observation) includes the presence of active giant papillae, gelatinous infiltrates of the limbus, exfoliative epithelial keratopathy, shield ulcer and papillary proliferation at lower palpebral conjunctiva; 2- the 10-point-grade group (10 points for each observation) includes the evaluation of blepharitis, papillary proliferation with velvety appearance, Horner-Trantas dots, edema of bulbal conjunctiva, and superficial punctate keratopathy; and 3- the 1-point-grade group (1 point for each observation) includes papillae at upper palpebral conjunctiva, follicular lesion at lower palpebral conjunctiva, hyperemia of palpebral conjunctiva, hyperemia of bulbal conjunctiva, and lacrimal effusion [45]. The total points in each grade group were determined as the severity score of the 5–5-5 exacerbation grading scale. Patients with VKC obtained the highest score compared to the ones suffering with allergic conjunctivitis or atopic keratoconjunctivitis. However, the main limitation of this scoring system is that it is not specific for VKC and it does not discriminate VKC from other allergic ocular disease. In recent studies, Shoji et al. evaluated the correlation between the clinical grading score previously proposed and levels of IL-16, Eosinophil Cationic Protein (ECP), CCL23 and other cytokine in tears [46, 47]. Remarkably, in children with the highest score in the 5–5-5 exacerbation grading scale for ocular allergic disease - especially those with VKC - IL-16, ECP and CCL23 were higher than the control group, showing a statistical significance. Similarly, Shiraki et al. measured CCL24 (Eotaxin-2) mRNA, one of the mediators of the allergic response, and compared the data obtained with clinical score, obtaining a positive and statistically significant correlation between laboratory findings and severity of VKC [33].

More recently, Gokhale at al. proposed a grading system based on severity of ocular signs, such as the presence of Horner-Trantas dots, cobblestone pattern, presence of superficial punctate keratitis and of micro- or macro-erosions, classifying the severity of signs into 4 categories: mild, moderate, severe and blinding [48]. Authors stressed the importance of considering the frequency of symptoms: patients with less of 4 episodes per year with complete remission of symptoms for more than 3–4 months suffer from an intermittent disease. Conversely, patients complaining ocular symptoms all around the year with a remission period less of 1 month can be considered affected by a chronic form of VKC. This grading score should be performed on both eyes at every visit in order to assess clinical finding and, more importantly, choose the best therapeutic approach accordingly.

Several grading schemes have been proposed also to estimate corneal involvement. The Oxford grading system is the most widely used in clinical trials and quantified epithelial surface damage in patients with dry eye according to a scheme of five panels which represent its severity, proportional to the amount of corneal staining [49]. Nevertheless, the Oxford is not specific for VKC and a modified Oxford scale was proposed, consisting of a seven-point scale with the addition of one grade between grade 0 and grade 1 and was used to evaluate corneal staining in patients with severe dry eye [50].

More recently Leonardi et al. proposed a new scoring system to better evaluate limbal and tarsal epithelial damage in patients with VKC. The new VKC-CLEK gives a more accurate evaluation in patients with limbal involvement. In this new scheme, the cornea and the limbal area are divided into five areas [51]. The total score is given by the sum of the staining scores assigned for each area, considering a score 0–4 for the central area and 0–1 for each limbal area. The total staining score is considered mild if less than 3, moderate if equal of more than 3 and less than 6 and severe if more than 6.

## Discussion

Today, we have numerous clinical scores available, some of which only built to evaluate the corneal damage. These grading scales are highly specialized and specific to the ophthalmologist who has to quantify the damage of the eye. Every ophthalmologist chooses the scale that best suits his clinical practice; assessment of merit on the superiority of a scale compared to another is beyond the pediatric skills.

There is no available attempt to integrate the ophthalmic scores with the clinical ones, except for the one proposed by Pucci et al. which is based on only subjective symptoms. It has the advantage of being easy and handy for the pediatrician, but also limits. The more consistent limitation is the lack of specificity that can lead to the potential underestimation of VKC, too easily confused with allergic conjunctivitis.

The Spadavecchia score is more specific while remaining simple and easy to apply. The limit consists in the scarce gradation of the severity of the disease. It is useful to establish that the disease is active when the score is > 3, but it could be more indicative if Authors had also established intermediate scores (between 3 and 8, maximum score) to create more levels of severity and to facilitate the choice of the therapeutic strategy. Bonini’s excellent attempt to create a better grading and a less fuzzy vision of the disease clashes with the need to formulate a very complex count, articulated in a mosaic of symptoms and situation. The result is a complex and difficult to apply scheme, especially for the pediatrician.

## Conclusions

There are no well-defined and uniform diagnostic criteria for VKC and no precise diagnostic criteria has yet been established. Moreover, no scoring system allows to share diagnostic criteria, management of the disease or therapy strategies.

The clinician (pediatrician) would need easy and agile tools to direct suspected VKC patients to the ophthalmologist and to agree and modulate the therapy. Only by increasing the knowledge on VKC and searching for possible surrogate markers of diagnosis and disease activity, it will be possible to state more homogeneous and specific scoring systems.

## Abbreviations

ECP: Eosinophil Cationic Protein
HMGB1: High-mobility group box-1
sRAGE: Soluble receptor for advanced glycation end products
VKC: Vernal keratoconjunctivitis
